# Monocyte-Derived Macrophages Expressing Dopamine D2-Subtype Receptors Drive Alcohol Effects on Mesolimbic Neurons and Microglia

**DOI:** 10.3390/biomedicines13102327

**Published:** 2025-09-23

**Authors:** Christina A. Nelson, J. Daniel Obray, Travis J. Clarke, James N. Brundage, Ryan J. Folsom, Carlos M. Moreno, Pacen E. Williams, Lauren H. Ford, Sandra Hope, K. Scott Weber, Kyle B. Bills, Jordan T. Yorgason, Scott C. Steffensen

**Affiliations:** 1 Department of Psychology and Center for Neuroscience, Brigham Young University, Provo, UT 84602, USA; casmall@noordacom.org (C.A.N.); h.laurenford@gmail.com (L.H.F.); 2 Department of Research, Noorda College of Osteopathic Medicine, Provo, UT 84606, USA; kbbills@noordacom.org; 3 Department of Microbiology and Molecular Biology, Brigham Young University, Provo, UT 84602, USA; cmoren22@student.byu.edu (C.M.M.);; 4 Department of Cellular Biology and Physiology, Brigham Young University, Provo, UT 84602, USA; jordan.yorgason@advocatehealth.org

**Keywords:** alcohol, microglia, macrophage, neuroinflammation, cytokines, neuroimmunomodulation, dopamine 2 receptors, monocytes

## Abstract

**Background/Objectives**: Microglia are the primary immune cells in the central nervous system (CNS) and are known as “resident” macrophages. The aim of this study was to determine the effect of acute ethanol (EtOH) on the microglia state and monocyte infiltration into the CNS, with particular attention to the role of peripheral and central dopamine (DA) D2 receptors (D2Rs) in mediating EtOH effects on peripheral and central substrates. We hypothesize that EtOH interacts with peripheral immune mediators via D2Rs including monocyte-derived macrophages (MDMs) to modulate midbrain neurons, DA transmission in the mesolimbic pathway from the ventral tegmental area (VTA) to nucleus accumbens (NAc), and the intoxicating effects of acute EtOH. **Methods**: Using the Macrophage FAS-Induced Apoptosis (MaFIA) mouse model (GFP+ on Csf1r promoter), we assessed the effects of three intraperitoneal (IP) doses of EtOH (1, 2, and 4 g/kg) at three time points (0.5, 1, and 2 h after injection) on D2R expression in blood leukocytes and microglia, as well as midbrain neuronal activity, DA release, and behavior. **Results**: Acute EtOH significantly enhanced lymphocyte and monocyte D2R expression at 1.0 g/kg by 2 h after injection in vivo but decreased D2R expression in vitro. Ethanol enhanced microglia D2R expression in the NAc, while not altering D2R expression in the VTA, but altered the microglia state in these areas, shifting them toward an inflammatory phenotype. Acute EtOH induced prolonged and progressive hypersensitivity of D2R activation of VTA GABA neurons. Intravenous injection of the macrophage depleter liposomal clodronate significantly reduced blood macrophages by 55.3% and blocked the typical inhibition of VTA GABA neurons by EtOH, as well as the enhancement of DA levels in the NAc, and the locomotor indices of intoxication produced by acute EtOH, but not choice place preference. **Conclusions**: These findings strongly suggest a neuroimmune peripheral connection for acute low-dose EtOH use and challenge the dogma that central actions of EtOH exclusively mediate its effect on DA neuronal activity and release.

## 1. Introduction

Alcohol use disorder (AUD) is the fourth leading preventable cause of death in the United States [1]. The underlying neural circuitry and various modulatory system interactions involved in AUD are incompletely characterized and this information is necessary for developing better treatments. Ventral tegmental area (VTA) dopamine (DA) projections to the nucleus accumbens (NAc) form a fundamental mesolimbic pathway for natural reward and motivation, including ethanol (EtOH) reinforcement [2]. These DA neurons are regulated by local circuit inhibitory γ-aminobutyric acid (GABA) neurons that are inhibited by EtOH, resulting in DA excitation (via disinhibition), which is thought to drive EtOH seeking behavior [3,4]. In vivo electrophysiology studies consistently show VTA neuron sensitivity to EtOH with an ED_50_ of 0.2 g/kg or ~5 mM [5,6]. In contrast, ex vivo studies on DA neurons demonstrate a relative insensitivity to EtOH, with an EC_50_ of 98.4 mM (Brodie et al., 1990) [7,8,9,10,11]. Likewise, we have shown that the VTA GABA neuron firing rate is inhibited by acute, low-dose EtOH in vivo [4,12,13,14,15], in contrast with relative insensitivity ex vivo [16,17]. Ethanol inhibits the excitability of VTA GABA neurons at physiologically relevant dose levels and alters GABAergic transmission onto DA neurons, providing evidence that they play a critical role in its rewarding properties [3,4]. Finally, DA terminals in accumbal brain slices show a similar lack of sensitivity for DA terminals to EtOH [18,19,20]. These discrepancies suggest the involvement of remote effects of EtOH that appear to be mediated peripherally. Accordingly, we recently demonstrated that some of EtOH’s effects on VTA neurons and DA release are mediated by peripheral DA D2 receptors (D2Rs) [21]. One possible source for increased sensitivity observed in vivo is peripheral leukocytes. We have shown that leukocytes express DA D2Rs [22], and our recent findings suggest that peripheral D2R activation contributes to central EtOH effects [21]. Further, EtOH has well-known peripheral immune effects that could contribute to reinforcement. For example, EtOH affects gut permeability to endotoxins such as LPS [23]. In turn, this can lead to the activation of peripheral blood mononuclear cells (PMBCs) including not just monocytes, B, T, and natural killer (NK) cells, but also resident macrophages including liver macrophages (Kupffer cells) and dendritic cells that release inflammatory cytokines such as IL-6, TNF-α, MCP-1, and IL-1β [24]. The blood–brain barrier (BBB) has transporters for cytokines, and these molecules may contribute to neuroinflammation [25,26].

Microglia are the primary immune cells in the central nervous system (CNS) and are known as the “resident” macrophages [27]. Derived from yolk sac progenitors that invade the embryo at embryonic day E9.5 and E10.5 [28], microglia have long been recognized for their roles in immune surveillance and response. While microglia were first characterized by del Rio-Hortega, their role has been shown to extend far beyond immunity. They play essential roles in the development of the nervous system through synaptic pruning [29] and have been implicated in a variety of neurodegenerative diseases such as Alzheimer’s disease [30], amyotrophic lateral sclerosis [31], and multiple sclerosis [32]. Microglia function is highly dynamic and context-dependent, influenced by signals in their environment. Traditionally, their activation states have been described using the M1/M2 framework where “M1” refers to a proinflammatory, classically activated state, and “M2” refers to anti-inflammatory or tissue repairing functions [33,34]. While this binary classification has been useful historically, it oversimplifies the diverse and plastic nature of microglial responses depending on the specific cues they encounter [35,36,37]. Microglial activation can be initiated by peripheral cytokines [27,38]. Chronic intermittent EtOH exposure can lead to the sensitization, or partial activation, of microglia through the upregulation of toll-like receptors (TLRs), priming them for further activation [39]. Toll-like receptor 4 (TLR4), in particular, has been shown to be integral to microglial activation by LPS and EtOH [40,41], and these are the same receptors activated by LPS on PMBCs.

Thus, we hypothesized that EtOH enhances peripheral DA levels, leading to increased D2R expression on monocyte-derived macrophages (MDMs), which in turn modulate mesolimbic microglia and related neurocircuitry. Through this peripheral-to-central immune signaling pathway, we propose that MDM activation via D2Rs contributes to changes in VTA GABA neuron activity, DA release in the NAc, and behavioral responses to acute EtOH. To test this hypothesis, we combined molecular, electrophysiological, neurochemical, and behavioral assessments with a targeted macrophage depletion approach using liposomal clodronate.

## 2. Materials and Methods

### 2.1. Animal Subjects

Male Wistar rats (>400 g) and both C57BL/6J wildtype mice and Csf1r-EGFP expressing macrophage FAS-induced apoptosis (MaFIA) mice on a C57BL/6J background (RRID:IMSR_JAX:005070; [42]) were obtained from a breeding colony at Brigham Young University and were used for visualizing microglia [43]. All animal procedures were carried out in accordance with the National Research Council’s Guide for the Care and Use of Laboratory Animals (2011) and were reviewed and approved by the Institutional Animal Care and Use Committee at Brigham Young University. Wistar rats used in this study were paired-housed. Mice were socially housed (groups of 2–5). Rats and mice were housed in separate temperature- and humidity-controlled rooms on a reversed schedule 12 h ON/12 h OFF light/dark cycle with lights off from 08:00 to 20:00 each day. All animals were given ad libitum access to food and water.

### 2.2. Flow Cytometry for Impact of In Vivo Ethanol on Lymphocyte and Monocyte Dopamine Subtype-2 Receptor Expression

For EtOH dose-response experiments, 8 rats were injected with saline and EtOH (1, 2, and 4 g/kg, IP). Injections were spread across two weeks and separated by a minimum of two days. Three h following each injection, rats were anesthetized using isoflurane (4%) and retro-orbital blood collection was performed. For EtOH time course experiments, 12 rats were injected with saline or EtOH (1 g/kg, IP) and underwent anesthesia and retro-orbital blood collection at 30, 60, and 120 min post-injection. Staining for D2R expression and flow cytometry were then carried out as previously reported [22]. In brief, 100 μL of whole blood was placed into 2 mL of 1X red blood cell lysis buffer (Biolegend, San Diego, CA, USA; 420301) for 10 min. Next, 2 mL of phosphate-buffered saline (PBS) was added and the samples were centrifuged at 2000 rpm for 5 min at 4 °C. The supernatant was then discarded and 2 mL of cell staining buffer was added. After incubating for 15 min on ice, the samples were again centrifuged and the supernatant was discarded. Subsequently, 2 mL of staining buffer containing rabbit anti-D2R antibody (1:1000; MilliporeSigma, Burlington, MA, USA; AB5084P) was added and samples were incubated for 15 min on ice. The samples were then centrifuged and the supernatant was discarded, after which staining buffer (2 mL) containing donkey anti-rabbit Alexa Fluor 750 (1:1000; Abcam, Cambridge, UK; ab175731) and FITC conjugated anti-CD45 (1:1000; BD Biosciences, San Jose, CA, USA; 554877) or T/B/NK kit antibodies (1:1000; BD Biosciences, San Jose, CA, USA, 558495) were added. Samples were then incubated for 15 min on ice and centrifuged. After the supernatant was discarded, 300 μL of cell staining buffer was added to each sample after which they were analyzed on a CytoFLEX flow cytometer (Beckman Coulter Life Sciences, Indianapolis, IN, USA). The acquired data were then evaluated using FlowJo V10 flow cytometry software (Becton, Dickinson and Company, Franklin Lakes, NJ, USA).

### 2.3. In Vitro Incubation of Lymphocytes and Monocytes and Expression of D2Rs

To examine the impact of EtOH and DA on lymphocyte and monocyte D2R expression, whole blood was obtained from 8 male Wistar rats by cardiac puncture. Equal parts whole blood and Hank’s balanced salt solution (HBSS) were mixed and layered onto lymphocyte separation media (Corning, NY, USA; 25-072-CI). The samples were then centrifuged (1650 rpm) in a refrigerated centrifuge (4 °C) for 15 min with no brake. The lymphocyte/monocyte layer was then extracted and added to 10 mL of HBSS. The samples were then centrifuged (1350 rpm, with brake) for 10 min. The resulting pellet was resuspended in 10 mL HBSS. Each sample was then dispersed into 24 wells and additional media containing EtOH (0 mM or 50 mM) and DA (0 nM, 10 nM, 100 nM, or 1 μM) was added. Incubation with each combination of EtOH and DA concentration was carried out in triplicate for every rat. Cells were then incubated (37 °C, 5% CO_2_) for 4 h. After incubating, the cells were manually harvested and stained for CD45 and D2R expression (see Section 2.2), with expression analyzed by flow cytometry.

### 2.4. Immunofluorescence Staining for Microglial Dopamine Subtype-2 Receptor Expression

Rats were deeply anesthetized (isoflurane, 4%) and then transcardially perfused with saline followed by 4% paraformaldehyde (PFA). Brains were then extracted and placed in 4% PFA for 48 h prior to being transferred to a 30% sucrose (*w*/*v* in PBS) in preparation for sectioning. Immediately prior to sectioning, brains were flash-frozen in a slurry of 2-methyl butane and dry ice. Accumbal and VTA sections were obtained with a freezing microtome (40 μm) and subsequently stored at −20 °C in cryoprotectant (30% *w*/*v* sucrose, 1% *w*/*v* PVP-40, and 30% *v*/*v* ethylene glycol in 1X PBS). For immunofluorescent labeling, tissue sections were first washed 3 times for 5 min each with 1X PBS. Tissue sections were next incubated in blocking solution (5% *v*/*v* normal donkey serum and 0.1% *v*/*v* Triton X-100 in 1X PBS) for 90 min. Following incubation in blocking solution, sections were washed 3X for 10 min each with 1X PBS after which sections were incubated in antibody dilution buffer (0.1% *w*/*v* bovine serum albumin (BSA) and 0.3% Triton X-100 in 1X PBS) containing primary antibodies rabbit anti-D2R (1:200; Millipore, gma, Burlington, MA, USA; AB5084P) and goat anti-IBA-1 (1:500; Abcam, Cambridge, UK; ab5076) overnight at 4 °C. The tissue was next washed 3X for 10 min each with 1X PBS followed by incubation in antibody dilution buffer containing secondary antibodies donkey anti-rabbit 594 (1:500; Abcam, Cambridge, UK; ab150076) and donkey anti-goat 488 (1:500; Abcam, Cambridge, UK; ab150129) for 2 h at room temperature. Finally, sections were washed 3X for 10 min each with 1X PBS before being mounted on microscope slides with Vectashield mounting media (Vector Laboratories, Newark, CA, USA; H-1400-10) and a coverslip placed on top. Microglia were imaged using a 20X objective on a Nikon (Tokyo, Japan) Eclipse Ti light microscope. To assess D2R expression, images were imported into ImageJ v1.54p [44] (NIH, Bethesda, MD, USA) and thresholded using the Shanbhag algorithm [45]. Following thresholding, a mask containing microglia was created. After creating the mask, the mean fluorescence intensity for the D2R expression on the microglia was obtained.

### 2.5. Confocal Microscopy for Microglia Volume-to-Surface-Area Ratio

Tissue slices from mice were obtained and mounted on slides as described in Section 2.4. Following mounting on slides, tissue sections were imaged under immersion oil at 40X using an Olympus FluoView FV1000 (Center Valley, PA, USA) confocal microscope. A z-step size of 1 μm and constant PMT voltage and gain were used between all acquired images. Once images were acquired, they were loaded into the image analysis program FIJI [44]. Images were then thresholded using the yen algorithm and rendered in 3D. The BoneJ v1.3.1 ImageJ v1.54p particle analyzer (NIH, Bethesda, MD, USA) was used to create meshes around each of the cells [46]. The surface area and enclosed volume were then measured. To determine the activation state, the ratio of the microglial volume to the surface area was determined, with an increase in this ratio indicating a change in the microglia state.

### 2.6. Isolation of Brain Microglia and PBMCs

Following isoflurane anesthetization, mice underwent transcardial perfusion with ice-cold PBS. Once perfused, brains were carefully removed from the skull and placed in ice-cold PBS and glued to a cutting stage. Accumbal and VTA horizontal slices (1 mm thick) were placed in an incubation chamber container containing PBS. Tissue dissections of the appropriate brain regions were then punched from the slices and transferred into 1.5 mL microcentrifuge tubes containing Roswell Park Memorial Institute medium (RPMI). The tissue was then ground in a 7 mL Dounce homogenizer containing 3.5 mL RPMI until the suspension was homogenous and RPMI was added to the suspension until the volume was 7 mL. Once homogenous, 3 mL 100% Percoll was added to the solution to create a final Percoll concentration of 30%. Homogenates were then carefully layered above a 3 mL solution of 70% Percoll in 15 mL conical tubes. Following layering, the samples were centrifuged for 30 min at 600 G with no brake. The different densities of the solutions caused PBMCs and microglia to move to the interphase between the 70% and 30% layers. The myelin and other debris were then carefully removed from the upper ~6 mL of solution, and the middle ~2 mL of the interphase was extracted and transferred to 5 mL round-bottom polystyrene tubes (FACS tubes) [47]. The samples were then centrifuged at 900 G at 4 °C. The supernatant was aspirated, and the pellet was resuspended in cell staining buffer (dissolve 8 g of NaCl, 0.2 g of KCl, 1.44 g of Na2HPO4, and 0.24 g of KH2PO4 in 800 mL distilled H_2_O, with 20 mL of heat-inactivated FBS.0.9 g of sodium azide, pH adjusted to 7.4). Then, to each sample, 2 μL of Fc Block (anti-CD32/CD16) (Leinco Technologies, St. Louis, MO, USA) was added. Each sample was gently mixed by flicking and incubated on ice for 15 min. After incubation, 0.5 μL of each primary antibody was added to all the experimental samples, but not to the unstained sample. Also, 100 µL of 1.0% BSA in 1X PBS was added to each sample. Then, the samples were incubated in the dark on ice for 30 min. The antibodies used for this experiment were anti-CD45 (FITC) (Life Technologies, ThermoFisher Scientific, Waltham, MA, USA), anti-CD11b (EF450) (Life Technologies, ThermoFisher Scientific, Waltham, MA, USA), and anti-CD40 (PE) (Life Technologies, ThermoFisher Scientific, Waltham, MA, USA). After incubation, 2 mL of 1.0% BSA in 1X PBS was added to rinse the non-bound antibody and then the samples were centrifuged at 2000 rpm for 5 min, decanted, and resuspended in residual fluid. Then, cold 1.0% BSA in 1X PBS was added to each sample to obtain a 200 μL dilute solution. Each sample was filtered through 70 μm mesh prior to flow use. Each individual test sample was prepared in FACS 12 mm × 75 mm plastic tubes.

### 2.7. Submandibular Blood Collection and Flow Cytometry in Clodronate Studies

Submandibular blood collection was performed under isoflurane anesthesia (4–5% until induction) with the mouse restrained. A lancet (5 mm) was used to puncture the submandibular vein. The lancet was held perpendicular to the bleed site to avoid puncturing the ear canal [48]. One hundred μL of blood was collected in a 1 mL microcentrifuge tube filled with 1.0% BSA in PBS. Gentle pressure with gauze was applied to the puncture site until bleeding stopped. A 100 μL aliquot of whole blood collected from the submandibular vein was placed in 2 mL of red blood cell lysis solution (BioLegend, San Diego, CA, USA) and gently vortexed and left to incubate in the dark for 15 min. The reaction was stopped with the addition of 2.0 mL PBS and centrifuged (2000 RPM) for 5 min. Afterwards, the supernatant was decanted, and the leftover pellet was resuspended in residual fluid by tapping. Then, 2 mL of 1.0% BSA in 1X PBS was added and the tube was gently vortexed. Then, the sample was centrifuged at 2000 RPM for 5 min and decanted, and the cells were resuspended in residual fluid by tapping. Then, 2 μL of Fc Block (anti-CD32/CD16) (Leinco Technologies, Fenton, MO, USA) was added. Each sample was gently mixed by flicking and incubated on ice for 15 min. After incubation, 0.5 μL of each primary antibody and 100 µL of 1.0% BSA in 1X PBS was added to all experimental samples while the unstained sample received only BSA in PBS. Next, the samples were incubated in the dark on ice for 30 min. The antibodies used were as follows: anti-CD45 (PECy7) (Invitrogen, ThermoFisher Scientific, Waltham, MA, USA), anti-F4/80 (APC) (Invitrogen, ThermoFisher Scientific, Waltham, MA, USA), and anti-D2R (PE) (Santa Cruz Biotech, Dallas, TX, USA). After incubation, 2 mL of 1.0% BSA in 1X PBS was added to rinse the non-bound antibody and then the samples were centrifuged at 2000 rpm for 5 min, decanted, and resuspended in residual fluid. Then, cold 1.0% BSA in 1X PBS was added to each sample to obtain a 200 μL dilute solution. Each sample was filtered through 70 μm mesh prior to flow use. Each individual test sample was prepared in FACS 12 mm × 75 mm plastic tubes. Samples for the clodronate experiments were analyzed using a BD Accuri C6 flow cytometer (BD Biosciences, San Jose, CA, USA) with two lasers for measuring up to four channels. Single and unstained controls were run in C57BL6/J mice and used to compensate the samples. Once samples were acquired in the flow cytometer, files were analyzed using FlowJo V10 flow cytometry software (Ashland, OR, USA).

### 2.8. In Vivo Single-Unit Recordings

Single-unit recordings of VTA GABA neurons were obtained from isoflurane (1.5–2.0%)-anesthetized Wistar rats (quinpirole activation studies) or C57Bl6/J mice (clodronate studies). Using an Inchworm 8200 microdrive (EXFO Burleigh) attached to a stereotaxic, micropipette electrodes (1–4 MΩ) loaded with 1 mg/mL quinpirole (Sigma Aldrich, Burlington, MA, USA; Q102) dissolved in a 1 M KCl solution were inserted into the VTA (for rats—from bregma: −5.3 to −5.7 mm AP, +0.6 to +1.0 mm ML, and −7.8 to −9.2 mm DV; for mice—from bregma: −2.7 to 3.0 mm AP, +0.3 mm ML, and −3.7 to −5.2 mm DV). Extracellular potentials were amplified using a Multiclamp 700A amplifier (Molecular Devices, San Jose, CA, USA). Single-unit firing was discriminated using a WP-121 spike discriminator (World Precision Instruments [WPI], Sarasota, FL, USA) and captured in 10 sec bins using a National Instruments (Austin, TX, USA) NB-MIO-16 digital I/O acquisition board connected to a Macintosh PC running custom LabVIEW v8 (National Instruments, Austin, TX, USA) software. Potentials were filtered at 300 Hz to 10 kHz (−3 db) and sampled at 20 kHz. Putative GABA neurons were classified based on previously established criteria including activation by iontophoresis of DA or D2R agonists, a short-duration action potential (<750 μs), and initially negative-going, non-bursting spikes [13,14,49,50,51].

For studies examining the effects of acute EtOH on the D2R-mediated activation of VTA GABA neurons, neuronal activity was recorded for a minimum of 30 min after which EtOH (16% *w*/*v*, 1–2 g/kg, IP, n = 16/group) or saline (n = 15) was administered. Recordings then continued for an additional 4 h post-injection. Quinpirole was applied iontophoretically (+30 nA ejection current and −10 nA retention current) for 1 min every 2 min for the duration of the experiment. The absolute effect of quinpirole on GABA neuron firing was obtained by identifying the median firing rate during each application of quinpirole and subtracting the median firing rate during the preceding 60 sec using an algorithm written in Python v3.9.7. To determine the effect of acute EtOH on the D2R-mediated activation of VTA GABA neurons, the average activation occurring prior to the injection was calculated and the activation in each bin was expressed as a percentage of the baseline activation.

For clodronate single-unit electrophysiology, baseline recordings of the firing rate were measured for 30 min before an injection of 0.75 g/kg IP EtOH (16% *w*/*v*). The firing rate was then recorded for an additional 120 min post-injection in 10 sec bins. To determine changes in the VTA GABA neuron firing rate produced by EtOH administration, the firing rate was determined by averaging 10 min epochs of activity before and at 10–20 min after EtOH injection.

### 2.9. Liposomal Clodronate Injection

Liposomal clodronate (10 µL/mg) was injected into MaFIA mice via the lateral tail vein. Tail vein injections were performed under 4% isoflurane anesthesia. The mice were restrained with a head stage and were warmed via heating pad. The tail was grasped between index and middle finger above the site of injection (digital pressure acting as a tourniquet). A 27-gauge needle was used to slowly inject the liposomes into the animal, releasing pressure from the proximal fingers before administering injectate. Vehicle liposomes were injected in the same manner and at the same dosage as the clodronate liposomes as a control [52].

### 2.10. Microdialysis and High-Performance Liquid Chromatography

On the test day, mice were anesthetized using isoflurane (4%) and microdialysis probes (BASI Technologies, Pasadena, CA, USA) were inserted into the NAc based on stereotaxic coordinates (from bregma: 1.2 mm anterior, 0.6 mm lateral, 4.5 mm ventral). Anesthesia was maintained at 1.5% with 2.0 L/min of air flow from a nebulizer driven by an oxygen concentrator. Body temperature was maintained at 37.0 ± 0.4 °C by a feedback-regulated heating pad. Artificial cerebral spinal fluid (aCSF) containing 148 mM NaCl, 2.7 mM KCl, 1.2 mM CaCl_2_, and 0.85 mM MgCl_2_ (final pH 7.4 by phosphoric acid) was perfused through the probe at a rate of 3.0 µL/min. During a baseline period beginning 20 min after probe insertion, microdialysis samples were collected every 20 min for 1 h. At the end of the baseline period, EtOH (0.75 g/kg, IP) was administered. Dialysate samples were then collected for an additional 2–3 h. Dialysate samples were stored in a freezer (−80 °C) to await analysis by HPLC. Determination of the DA content in the dialysate was performed using a high-performance liquid chromatography (HPLC) pump (Ultimate 3000, Dionex, Sunnyvale, CA, USA) connected to a coulometric detector (Coulochem III, ESA, Cerritos, CA, USA). The coulometric detector included a guard cell (5020, ESA) set at +275 mV, a screen electrode (5014B, ESA) set at −100 mV, and a detection electrode (5014B, ESA) set at +220 mV. Dopamine was separated using a 2.1 × 50 mm PA2 reverse phase column (2.2 µm particle size; Acclaim, Life Technologies, Thermo Fisher Scientific, Waltham, MA, USA). The mobile phase containing 150 mM NaH2PO4, 4.76 mM anhydrous citric acid, 3 mM sodium dodecyl sulfate (SDS), 50 μM ethylenediaminetetraacetic acid, 15% *v*/*v* acetonitrile, and 10% *v*/*v* methanol (final pH = 5.6 by NaOH) was pumped through the system at a flow rate of 0.4 mL/min [53]. The detection limit for DA using this setup was 95 pg/mL. External standards (500 pm and 5 nm) were assayed concurrently with the samples allowing for the construction of a calibration curve using Chromeleon software (v7.2, Thermo Fisher Scientific, Waltham, MA, USA). This curve was then used to provide estimates of the DA concentration in the samples. Dopamine levels following drug administration were expressed as a percentage of the baseline DA levels. Baseline DA levels were computed as the average DA concentration collections occurring prior to drug administration.

### 2.11. Behavioral Procedures

Locomotor trials lasted for 30 min. All subjects received injections of saline (isovolumic to EtOH) or EtOH (2.0 g/kg, IP). Mice were immediately placed into the chamber following the injection. The open-field cage consisted of two clear-walled cages (17 × 9 × 8 in.). The mouse was placed in the center of the cage and allowed to move freely for 30 min while a camera recorded movement. Recordings were later evaluated by two independent reviewers using MATLAB vR2024 (MathWorks, Natick, MA, USA) software. Videos were evaluated for total distance traveled (m), and ratio of time spent in the inner/outer areas of the cage (thigmotaxis).

Place conditioning was conducted using a modified version of a one-chamber paradigm previously shown to induce ethanol place preference in C57Bl/6J mice [54]. Mice (n = 24) were randomly assigned to one of four treatment groups: (1) saline/saline + control liposomes, (2) saline/saline + clodronate liposomes, (3) saline/ethanol (2 g/kg, IP) + control liposomes, or (4) saline/ethanol (2 g/kg, IP) + clodronate liposomes. The conditioning apparatus measured 40.6 × 40.6 × 40.6 cm. During pre-test and post-test trials, smooth and rough floor panels each covered half the chamber floor (824 cm^2^ each). During conditioning trials, the full floor (1648 cm^2^) was covered by a single panel, alternating between smooth and rough textures. A biased design was used: the initially unpreferred texture was paired with treatment. The procedure consisted of three phases: a 30 min pre-test to assess initial preference, eight 5 min conditioning sessions (twice daily for four days), and a 30 min post-test to assess conditioned preference. Liposomal clodronate or control liposomes were administered immediately after the pre-test and after the second conditioning session. For saline/saline mice, the treatment context was defined as the one from which they were removed just before the second liposome injection. For ethanol-treated mice, the treatment context was the ethanol-paired texture. Ethanol or saline was administered immediately before each conditioning session. Ethanol sessions were always conducted in the afternoon to allow for drug clearance between sessions. To facilitate cross-study comparisons, time spent in each context is reported in s/min. An ANOVA was used to compare changes in preference scores (post-test—pre-test) across groups. A repeated measures ANOVA assessed whether preference for the treatment context changed between pre-test and post-test.

### 2.12. Drugs and Chemicals

Ethanol was diluted to 16% *w*/*v* using 0.9% sodium chloride and injected at volumes of 3.1–25 mL/kg depending on the dose of EtOH being administered.

### 2.13. Data Analysis and Statistics

Discriminated VTA GABA neuron spikes and stimulation events were processed with National Instruments LabVIEW v8 (National Instruments, Austin, TX, USA) and IGOR Pro software (Wavemetrics, Lake Oswego, OR, USA). A multilevel model was used to determine the significance of acute EtOH effects on the quinpirole-induced activation of VTA GABA neurons. To determine changes in the VTA GABA neuron firing rate produced by EtOH administration, the firing rate was determined by averaging 10 min epochs of activity before and at 10 min after EtOH injection. The results for control and drug treatment groups were derived from calculations performed on ratemeter records and are expressed as means ± S.E.M. Analysis of variance (ANOVA) was used to compare group means in microscopy and flow cytometry experiments. To adjust for multiple comparisons, a Dunnett’s post hoc test was used to compare treatment groups to control groups. Measures of effect size are reported for *p*-values less than 0.05, with the effect size 95% confidence interval included in brackets. Behavior experiments were used for clodronate studies to provide commentary on effect in lieu of η^2^. The statistical program R v3.5.0 (R Foundation, Vienna, Austria) was used to analyze microscopy data, JMP13 (Cary, NC, USA) was used for analysis of flow cytometry data, and Stata v15 College Station, TX, USA), was used for electrophysiological and behavioral data. Figures were constructed in Igor Pro v9.05 (Wavemetrics, Lake Oswego, OR, USA).

## 3. Results

### 3.1. Acute Ethanol Enhances Lymphocyte and Monocyte D2R Expression In Vivo

**Figure 1:**
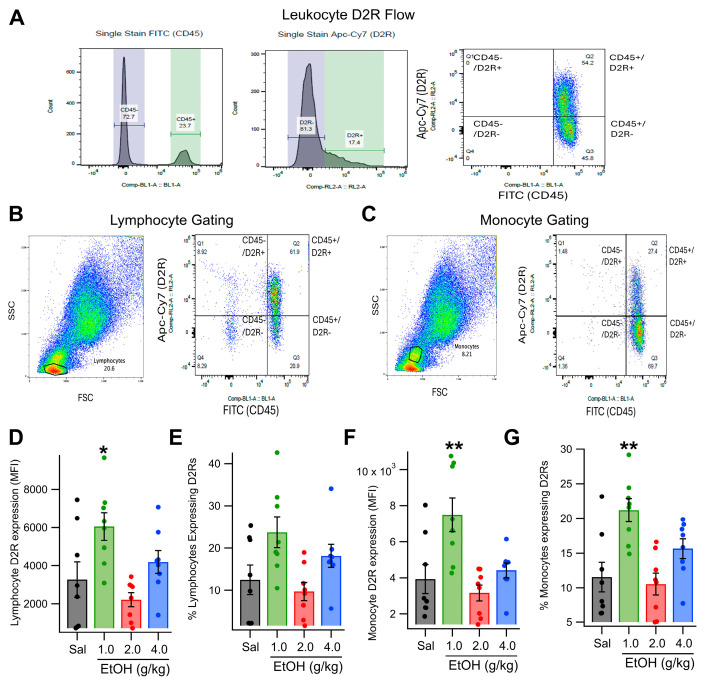
Effects of Acute EtOH on Lymphocyte and Monocyte D2R Expression: Dose Response. (**A**) The gating scheme for evaluating D2R expression in leukocytes with flow cytometry. (**B**,**C**) Lymphocyte vs. monocyte gating with flow cytometry. Side scatter (SSC) is plotted against forward scatter (FSC; left graph) and gates for lymphocytes and monocytes are determined based on standards for FSC vs. SSC graphs. Right graphs show CD45 against D2R to determine D2R expression on the subpopulations (lymphocytes or monocytes). (**D**–**G**) Intraperitoneal administration of EtOH (1.0–4.0 g/kg) had complex effects on blood lymphocyte and monocyte D2R expression. (**D**,**E**) Ethanol increased D2R expression in lymphocytes and exhibited a trend toward increased number of lymphocytes expressing D2Rs at 1.0 g/kg EtOH. (**F**,**G**) Similarly, EtOH increased D2R expression in monocytes and increased the number of monocytes expressing D2Rs at 1.0 g/kg. Asterisks *, ** represent significance levels *p* < 0.05 and *p* < 0.01, respectively.

It is well known that prolonged changes in central DA signaling result in altered expression of D2Rs [55,56,57]. Experiments conducted in vitro have further confirmed this by demonstrating DA-dependent changes in D2R expression [58,59,60]. However, the effects of changes in blood DA levels on D2R expression on leukocytes are less well studied, despite reports of altered leukocyte D2R expression in several diseases involving DA dysfunction [61,62]. Similar to what is observed with changes in central DA systems, we recently demonstrated that incubation of human leukocytes in DA enhances D2R expression in lymphocytes in restless legs disorder (RLS) [22]. To extend these findings, the first set of experiments used flow cytometry to examine the effect of different doses of acute EtOH, some of which enhance plasma DA levels [21] on lymphocyte and monocyte D2R expression (Figure 1). To accomplish this, both the percentage of D2R-expressing cells and the mean fluorescence intensity (MFI) of D2R staining were quantified (Figure 1A–C). The analysis revealed a dose-dependent increase in D2R MFI for both lymphocytes (main effect dose: *F*_(3, 21)_ = 5.68, *p* = 0.0174, partial η^2^ = 0.4480 [0.0633, 0.6081]; Figure 1D) and monocytes (main effect dose: *F*_(3, 21)_ = 13.96, *p* = 0.0012, partial η^2^ = 0.6661 [0.3201, 0.7671]; Figure 1F) 120 min following EtOH administration.

A Dunnett’s test comparing D2R expression after each dose of EtOH to expression following a control saline injection revealed that only the 1.0 g/kg dose of EtOH significantly enhanced D2R expression in lymphocytes (*t* = 2.88, *p* = 0.024) and monocytes (*t* = 4.96, *p* < 0.001). The percentage of lymphocytes that expressed D2Rs was unaltered by EtOH (main effect dose: *F*_(3, 21)_ = 3.30, *p* = 0.0783; Figure 1E). In contrast, the percentage of monocytes expressing D2R was increased significantly by EtOH (main effect dose: *F*_(3, 21)_ = 7.94, *p* = 0.0058, partial η^2^ = 0.5315 [0.1429, 0.6706]; Figure 1G). Dunnett’s test revealed that only the 1.0 g/kg dose of EtOH increased the percentage of monocytes expressing D2Rs relative to the saline control (*t* = 3.97, *p* = 0.002).

**Figure 2:**
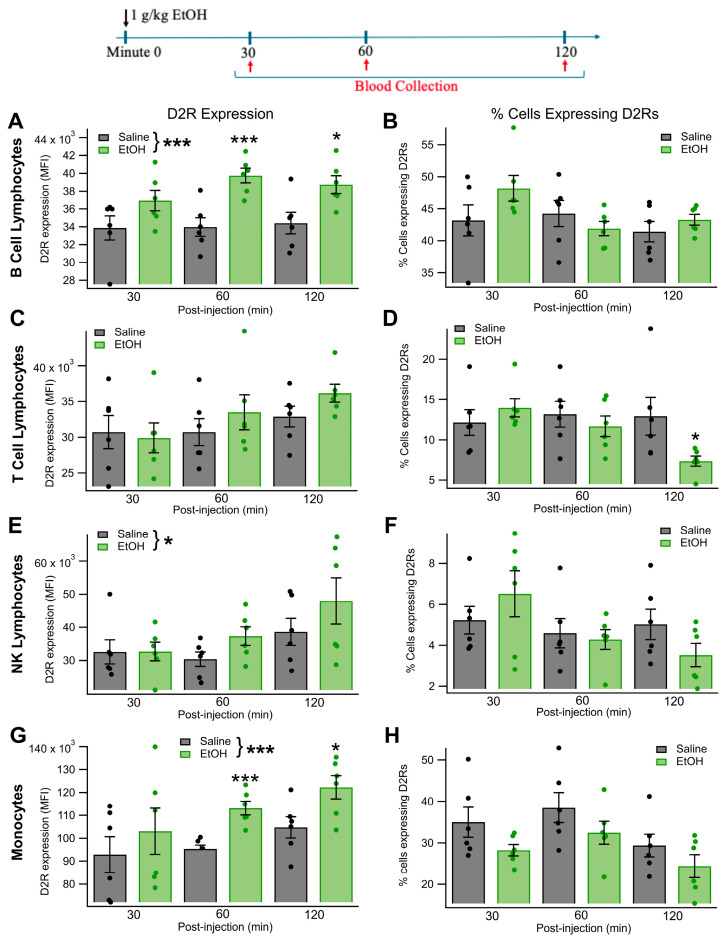
Effects of Acute EtOH on Lymphocyte and Monocyte D2R Expression in Specific Lymphocyte Populations: Time Course: Given that most of the reactivity was at 1.0 g/kg EtOH (Figure 2), we followed D2R MFI expression in monocytes and subpopulations of lymphocytes for 2 h after injection. We evaluated three populations of lymphocytes: B cells, T cells, and natural killer (NK) cells. The graphical illustration at the top shows the method involved in this experiment; mainly, that a dose of EtOH was chosen for time course studies based on the outcome of the dose-response studies in Figure 1, but sampled at three time points after injection of EtOH at 1.0 g/kg. (**A**,**B**) In B cell lymphocytes, 1.0 g/kg EtOH significantly increased B cell lymphocytic D2R expression (MFI) but not the number of cells expressing D2Rs. (**C**,**D**) In T cell lymphocytes, 1.0 g/kg EtOH had no significant effects on D2R expression or on the # of cells expressing D2Rs. (**E**,**F**) In NK cell lymphocytes, 1.0 g/kg EtOH had no significant effects on D2R expression or on the number of cells expressing D2Rs. (**G**,**H**) In monocytes, 1.0 g/kg EtOH significantly increased monocyte D2R expression, but mostly at the 60 and 120 min time points. It did not significantly affect the number of cells expressing D2Rs. Asterisks *, *** represent significance levels *p* < 0.05 and *p* < 0.001, respectively.

After assessing the effects of different doses of EtOH on monocyte and lymphocyte D2R expression, we then sought to characterize the time course of changes in D2R expression following an acute dose of EtOH and with attention to different types of lymphocytes. As the 1.0 g/kg dose of EtOH produced the most robust changes in lymphocyte and monocyte D2R expression (Figure 1), blood samples were taken 30, 60, and 120 min following an IP injection of either EtOH (1.0 g/kg, IP) or saline (equivolumic). The level of D2R MFI and the percentage of cells expressing D2R were quantified for monocytes, B, T, and NK lymphocytes. For B cells, D2R expression levels (MFI) were elevated following EtOH administration (main effect EtOH: *F*_(1, 10)_ = 22.45, *p* = 0.0008, partial η^2^ = 0.6919 [0.2218, 0.8249]; Figure 2A). There was not a significant effect of time (main effect time: *F*_(2, 20)_ = 1.02, *p* = 0.3650) or an interaction between time and EtOH on B cell D2R expression (EtOH × time interaction: *F*_(2, 20)_ = 0.78, *p* = 0.4452; Figure 2B). The percentage of B cells expressing D2Rs was unaltered by EtOH administration (main effect EtOH: *F*_(1, 10)_ = 1.21, *p* = 0.2979) and was stable over time (main effect time: *F*_(2, 20)_ = 1.91, *p* = 0.1800) with no interaction between EtOH and time (EtOH × time interaction: *F*_(2, 20)_ = 2.12, *p* = 0.1535). For T lymphocytes, D2R MFI was unchanged following EtOH administration (main effect EtOH: *F*_(1, 10)_ = 0.81, *p* = 0.3884; Figure 2C), remained stable over time (main effect time: *F*_(2, 20)_ = 3.08, *p* = 0.0684), and did not display a significant interaction between EtOH and time (EtOH × time interaction: *F*_(2, 20)_ = 0.87, *p* = 0.4353; Figure 2D). In contrast, while the percentage of T cells expressing D2Rs was unchanged by EtOH (main effect EtOH: *F*_(1, 10)_ = 1.15, *p* = 0.3085) or time alone (main effect time: *F*_(2, 20)_ = 3.14, *p* = 0.0651), acute EtOH decreased the percentage of D2R-expressing T cells in a time-dependent manner (EtOH × time interaction: *F*_(2, 20)_ = 4.60, *p* = 0.0278, partial η^2^ = 0.3151 [0.0027, 0.5260]). Specifically, there was a trend toward a reduced percentage of D2R-expressing T cells in EtOH-treated rats 120 min post-injection (*t* = −2.58, *p* = 0.053; Figure 2D). For NK lymphocytes, MFI was elevated following EtOH administration (main effect EtOH: *F*_(1, 10)_ = 7.24, *p* = 0.0226, partial η^2^ = 0.4200 [0.0037, 0.6687]; Figure 2E).

There was not a significant effect of either time (main effect time: *F*_(2, 20)_ = 3.13, *p* = 0.0855) or a time-dependent effect of EtOH (EtOH × time interaction: *F*_(2, 20)_ = 0.53, *p* = 0.5469; Figure 2F) on NK cell D2R expression levels. The percentage of NK cells expressing D2Rs did not change following EtOH administration (main effect EtOH: *F*_(1, 10)_ = 0.04, *p* = 0.8423). However, the percentage of D2R-expressing NK cells did vary over time (main effect time: *F*_(2, 20)_ = 4.82, *p* = 0.0499, partial η^2^ = 0.3254 [0.0072, 0.5342]; Figure 2F). This change occurred independently of whether the rat received an EtOH or saline injection (EtOH × time interaction: *F*_(2, 20)_ = 3.06, *p* = 0.1031). For monocytes, D2R MFI was elevated following EtOH administration (main effect EtOH: *F*_(1, 10)_ = 9.91, *p* = 0.0104, partial η^2^ = 0.4978 [0.0386, 0.7147]; Figure 2G). There was not a significant effect of time (*F*_(2, 20)_ = 3.19, *p* = 0.0919) or an interaction between time and EtOH (*F*_(2, 20)_ = 0.24, *p* = 0.6842) on monocyte D2R expression (Figure 2H). In contrast, the percentage of D2R-expressing monocytes was unaffected by EtOH administration (main effect EtOH: *F*_(1, 10)_ = 3.07, *p* = 0.1103), but did vary over time (main effect time: *F*_(2, 20)_ = 8.85, *p* = 0.0032, partial η^2^ = 0.4696 [0.1009, 0.6426]; Figure 2H), independent of EtOH treatment status (EtOH × time interaction: *F*_(2, 20)_ = 0.09, *p* = 0.8824).

### 3.2. Dopamine Receptor 2 Expression in Monocytes, but Not Lymphocytes, Is Sensitive to Incubation in Dopamine and Ethanol In Vitro

**Figure 3:**
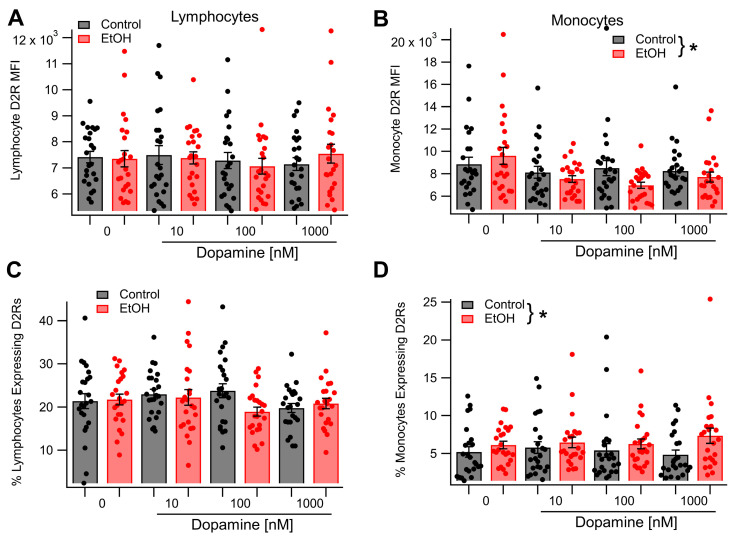
Effects of Dopamine and Ethanol on Lymphocyte and Monocyte D2R Expression In Vitro: (**A**,**C**) Neither EtOH (50 mM) nor DA (10–1000 nM) had effects on lymphocyte D2R expression or on the number of lymphocytes expressing D2Rs. (**B**,**D**) Ethanol in the presence of DA decreased monocyte D2R expression, but increased the number of cells expressing D2Rs. Asterisk * represents significance level *p* < 0.01.

Having demonstrated that acute EtOH enhanced D2R expression in lymphocytes and monocytes in vivo, we next sought to determine whether EtOH enhancement of D2R expression resulted from direct effects of EtOH on monocytes and lymphocytes or if it involved additional signaling factors. To accomplish this, leukocytes were incubated for 4 h in cell culture media either in the presence or absence of 50 mM EtOH. Cells were then harvested and underwent immunostaining for D2Rs. Dopamine receptor 2 expression levels were quantified using flow cytometry to determine both the percentage of cells expressing D2Rs and the density of D2R expression. For lymphocytes, both the density of D2R expression (*t*_(46)_ = 0.1619, *p* = 0.8721) and the percentage of cells expressing D2Rs (*t*_(46)_ = −0.17, *p* = 0.86) were unchanged following incubation in EtOH. Similarly, for monocytes, both the density of D2R expression (*t*_(46)_ = −0.76, *p* = 0.45) and the percentage of cells expressing D2Rs (*t*_(46)_ = −1.09, *p* = 0.28) were unaffected by incubation in EtOH. Previous research has demonstrated that incubation of D2R-expressing cells in media containing DA causes upregulated D2 expression [1,2,3,4]. As EtOH enhances plasma DA levels in vivo [5], it was hypothesized that incubation of leukocytes in media containing both EtOH and DA would recapitulate the upregulation of D2R expression observed in vivo following an acute dose of EtOH. The next experiment tested this hypothesis. Leukocytes were incubated for 4 h in cell culture media containing DA at one of three concentrations (10 nM, 100 nM, or 1 µM). Half of the samples at each concentration of DA also contained 50 mM ethanol. After incubation, the cells were harvested and underwent immunostaining and flow cytometry to determine the density of D2R expression and the percent of lymphocytes or monocytes expressing D2Rs (Figure 3). For lymphocytes, the density of D2R expression was unaffected by incubation in EtOH (main effect EtOH: *F*_(1, 137)_ = 0.01, *p* = 0.92), the concentration of DA (main effect DA concentration: *F*_(2, 137)_ = 0.40, *p* = 0.67), or the interaction between EtOH and DA concentration (EtOH × DA concentration interaction: *F*_(2, 137)_ = 0.58, *p* = 0.56; Figure 3A). The percentage of D2-expressing lymphocytes was similarly unaltered following incubation in EtOH (main effect EtOH: *F*_(1, 137)_ = 1.92, *p* = 0.17). Additionally, there was no effect of the concentration of DA (main effect DA concentration: *F*_(2, 137)_ = 1.47, *p* = 0.23) or an interaction between EtOH and the concentration of DA (EtOH × DA concentration interaction: *F*_(2, 137)_ = 2.52, *p* = 0.08) on the percentage of lymphocytes expressing D2Rs (Figure 3C). For monocytes, the density of D2R expression was reduced by EtOH when incubated in the presence of DA (main effect EtOH: *F*_(1, 137)_ = 5.45, *p* = 0.0211, partial η^2^ = 0.04 [0.004, 0.118]; Figure 3B). However, D2R expression was not altered by DA in a dose-dependent manner (main effect DA concentration: *F*_(2, 137)_ = 0.14, *p* = 0.87) and the effect of EtOH on D2R expression was not dependent on the concentration of DA in the culture media (*F*_(2, 137)_ = 0.71, *p* = 0.49). In contrast, the percentage of monocytes expressing D2Rs was increased by EtOH in the presence of DA (main effect EtOH: *F*_(1, 137)_ = 4.49, *p* = 0.036, partial η^2^ = 0.032 [0.0, 0.107]; Figure 3D), but was not affected by the concentration of DA (main effect of DA concentration: *F*_(2, 137)_ = 0.08, *p* = 0.93). The effect of EtOH on the percentage of monocytes expressing D2Rs was not found to be dependent on the concentration of DA present in the culture media (*F*_(2, 137)_ = 0.80, *p* = 0.45).

### 3.3. In Vivo Acute Ethanol Modulates Microglial D2R Expression

Although decreased striatal D2R expression has been observed following chronic EtOH misuse [63,64], little is known about how acute EtOH affects mesolimbic D2R expression. As such, the next study examined the impact of acute EtOH on central D2R expression. To accomplish this, either saline (n = 4) or acute EtOH (2.0 g/kg, ip, n = 8) was administered, after which the rats were euthanized, and their brains fixed and removed for IHC at 120 min post-injection. The brains were subsequently sliced and stained with antibodies against NeuN, IBA-1, and D2R.

**Figure 4:**
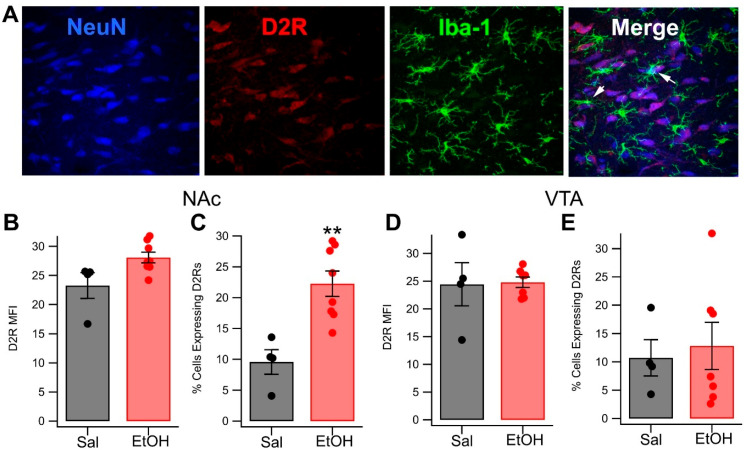
Effects of Acute EtOH on Microglia D2R Expression: (**A**) Representative images of D2R expression in neurons (NeuN) and microglia (Iba-1) in the VTA. Co-expression was evident for most DA neurons and some microglia in the merge. (**B**,**C**) Intraperitoneal administration of 2.0 g/kg EtOH had no effects on microglial D2R MFI in the NAc, but significantly enhanced the number of accumbal microglia expressing D2Rs. Ethanol did not affect microglial D2R expression in the VTA (**D**,**E**). Asterisks ** represents significance level *p* < 0.01.

The level of D2R expression on microglia in the NAc and VTA was then quantified (Figure 4). Ethanol had no effect on the density of microglial D2R expression in the NAc (*t*_(4.8)_ = −2.0125, *p* = 0.1032), but significantly increased the number of cells expressing D2Rs (*t*_(11.1)_ = −4.4413, *p* = 0.0010, Hedges’s *g* = −2.1977 [−3.5973, −0.7397]; Figure 4B,C), but there was no effect in the VTA (MFI: *t*_(3.6)_ = −0.0944, *p* = 0.9298; percentage of microglia expressing D2R: *t*_(11.0)_ = −0.3996, *p* = 0.6971; Figure 4D,E).

### 3.4. In Vivo Acute Ethanol Leads to the Activation of Mesolimbic Microglia

**Figure 5:**
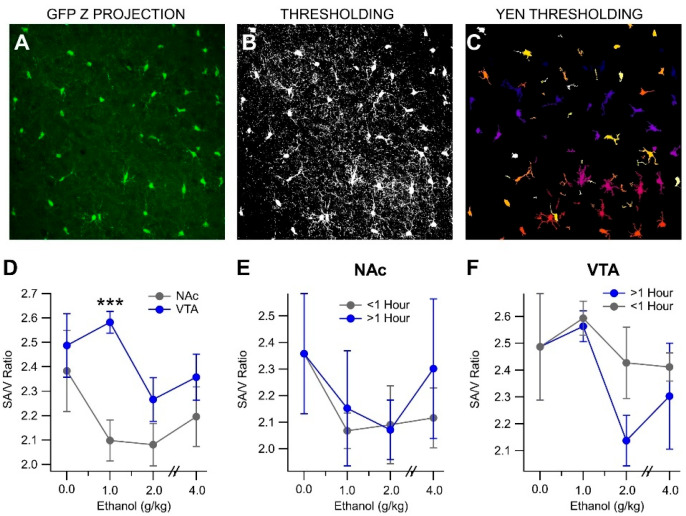
Effects of Acute EtOH on Microglia Morphology: (**A**) A sample z projection of MaFIA microglia taken by the microscope. (**B**) Image thresholded. (**C**) Yen thresholded. (**D**) There were significant differences in microglial surface area/volume (SA/V) ratio between the NAc and VTA after acute 1.0 g/kg EtOH with a biphasic response in the NAc, but mostly inhibition of SA/V ratio in the VTA. (**E**) However, EtOH did not significantly change SA/V ratio of microglia in the NAc or VTA (**F**). Asterisks *** represent significance level *p* < 0.001.

Postmortem studies have indicated an increase in microglial activation markers such as IBA-1 and GLUT-5 in chronic EtOH drinkers [65]. To determine if acute EtOH induced changes in microglia morphology, we imaged microglia in the NAc and VTA of MaFIA mice injected with EtOH (1, 2, or 4 g/kg, ip) and euthanized at 0.25, 0.5, 1, or 2 h post-injection. The middle 18 µm of the brain slices were imaged in 1 µm steps using a confocal microscope (see Supplemental Video S1 showing 3D microglia structure in the VTA with Iba-1 and NeuN immunolabeling). Changes in the surface area and enclosed volume of microglia were grouped by time (<1 h and >1 h) and analyzed (Figure 5A–C).

It was found that the surface-area-to-volume ratio was region-dependent. A significant difference in the SA/V ratios between the VTA and NAc was observed with 1 g/kg EtOH (*t*_(26)_ = 2.055, *p* < 0.0001; Figure 5D). However, in an analysis of EtOH-dependent effects in both NAc and VTA, no significant differences in the SA/V ratios were found at either time point and all doses (NAc (<1 h): *F*_(3, 17)_ = 0.930, *p* = 0.4475; NAc (>1 h): *F*_(3, 12)_ = 0.415, *p* = 0.7451; VTA (<1 h): *F*_(3, 16)_ = 0.651, *p* = 0.594; VTA (>1 h): *F*_(3, 16)_ = 2.404, *p* = 0.114; Figure 5E,F).

### 3.5. In Vivo Acute Ethanol Alters Microglia Polarization

**Figure 6:**
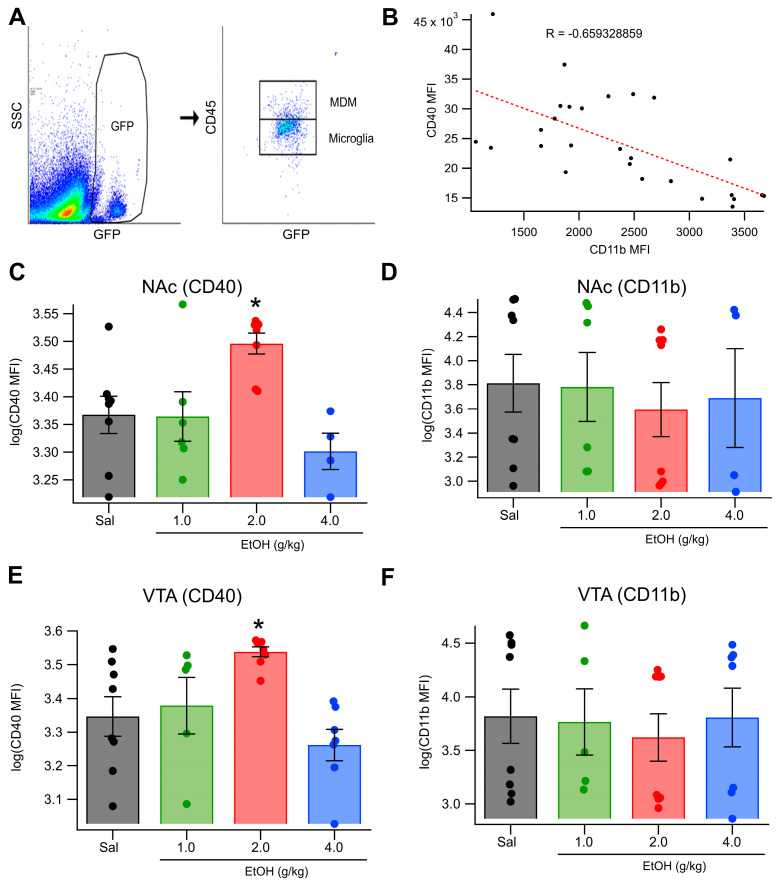
Effects of Acute EtOH on Microglia Phenotype: (**A**) The gating scheme for finding monocyte-derived macrophages. Cells that were GFP+/CD45hi/CD11bhi were considered MDMs. (**B**) A scatterplot comparison of marker CD40 and CD11b showing a negative correlation between MFI of the two proteins. (**C**) Intraperitoneal EtOH significantly increased CD40 (M1 phenotype) microglial expression in the NAc, but only at 2.0 g/kg. (**D**) EtOH did not significantly reduce expression of CD11b in NAc microglia at the same dose level. (**E**) Similarly, EtOH increased expression of CD40 microglia expression in the VTA, but only at the 2.0 g/kg dose level. (**F**) EtOH did not significantly decrease CD11b expression in the VTA. Asterisk * represents significance level *p* < 0.05.

Microglia function is influenced by their activation state, which has historically been categorized along a pro- vs. anti-inflammatory axis [33]. In the present study, we used CD40 and CD11b expression levels to characterize microglial activation states, reflecting the prevailing classification framework at the time these experiments were conducted. While newer approaches offer more nuanced interpretations of microglial function [66], the markers used remain informative for distinguishing pro- vs. anti-inflammatory tendencies in microglia. CD40 is commonly associated with inflammatory, “classically activated” microglia, while CD11b has been linked to more homeostatic and regulatory functions [34,35]. We investigated the microglial responses to acute EtOH (1, 2, or 4 g/kg; IP) at 1 r (1 g/kg EtOH) or 2 h (2, 4 g/kg) post-injection in MaFIA mice. Microglia were stained with antibodies targeting CD40, CD11b, and CD45 (pan-leukocyte marker) and analyzed using a CytoFlex flow cytometer. Cells that were GFP+/CD45hi/CD11bhi were considered MDMs (Figure 6A). Data were normalized across cohorts using z-scores of mean fluorescent intensity (MFI). In the NAc, IP injection of EtOH significantly increased CD40 expression (F_(3, 22)_ = 6.026, *p* = 0.00371; Figure 6C) but did not significantly affect CD11b expression (Figure 6D). In the VTA, IP EtOH also significantly increased CD40 expression (F_(3, 24)_ = 7.6468, *p* = 0.00093; Figure 6E), but did not significantly alter CD11b expression (Figure 6F). Post hoc analysis showed that 2 g/kg EtOH resulted in the greatest increase in CD40 in both the NAc and VTA (NAc: *t*_(7)_ = 3.35, *p* = 0.0061; VTA: *t*_(7)_ = 4.385, *p* = 0.0016; Figure 6C,E). To ensure that the effect was not an artifact of the staining or autofluorescence, we compared the CD11b and CD40 fluorescence intensities for each cell, and we found that CD11b and CD40 were negatively correlated (Figure 6B, −0.659). Thus, acute intoxicating doses of EtOH caused microglia to polarize toward an inflammatory phenotype.

### 3.6. Ethanol Enhances D2R-Dependent Activation of VTA GABA Neurons over a Protracted Period

**Figure 7:**
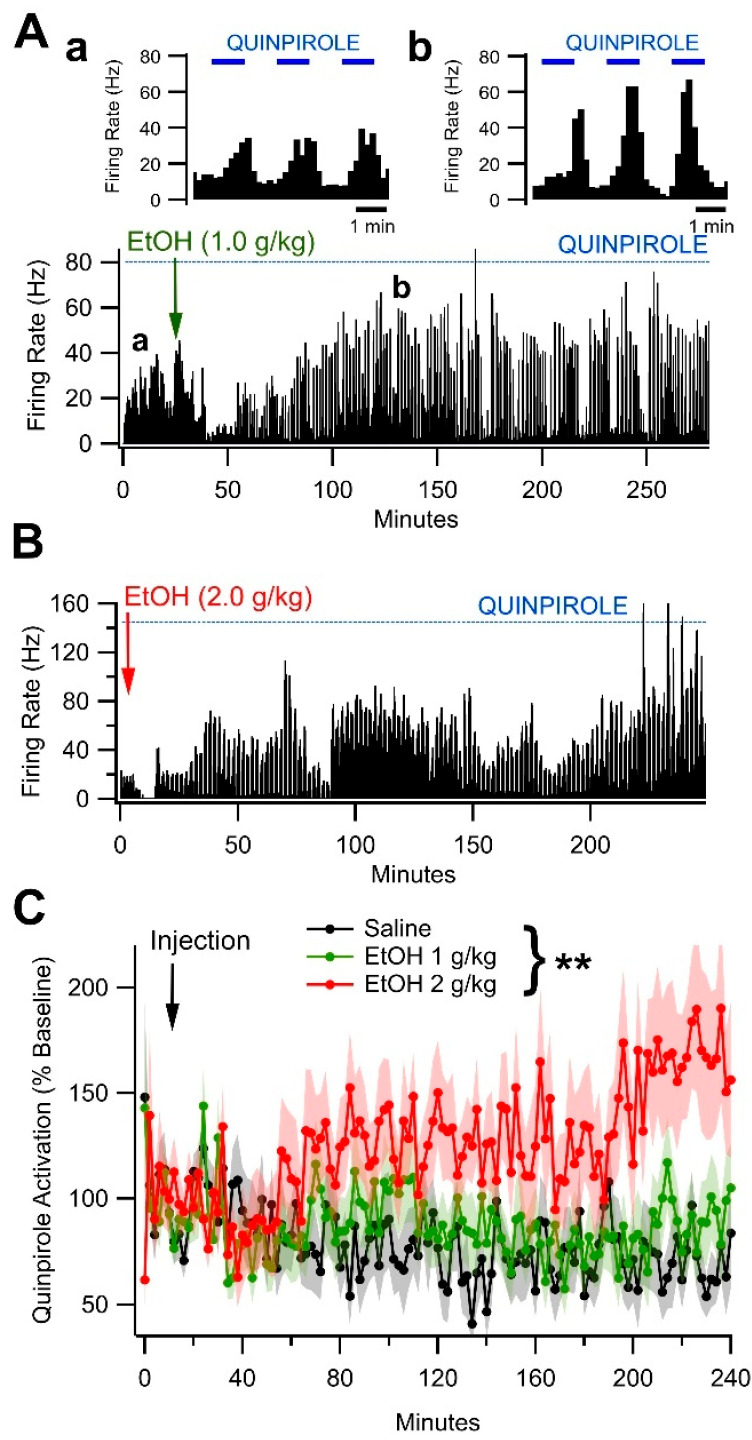
Effects of Acute EtOH on Quinpirole D2R-mediated Activation of VTA GABA Neurons: (**A**) Insets (**a**,**b**) show representative epochs of quinpirole in situ microintophoresis before and after 1.0 g/kg EtOH at the time points indicated on the graph below. The baseline firing rate of this neuron was approximately 20 Hz. Microelectrophoretic quinpirole markedly enhanced the firing rate of this VTA GABA neuron, which was repeatable, with little or no acute sensitization or tolerance. Intraperitoneal administration of 1.0 g/kg EtOH modestly decreased the firing rate of this VTA GABA neuron, decreased quinpirole activation at 20–30 min, but markedly enhanced quinpirole activation at 1–4 h after IP administration. (**B**) This ratemeter record shows the effects of 2.0 g/kg EtOH on quinpirole activation of the firing rate of a VTA GABA neuron in a separate experiment. The baseline firing rate of this neuron was approximately 18 Hz. Intraperitoneal administration of 2.0 g/kg EtOH suppressed the firing rate of this VTA GABA neuron, decreased quinpirole activation at 20–30 min, but markedly enhanced quinpirole activation at 1–4 h after IP administration. Horizontal bars indicate time of quinpirole current ejection from the micropipette. (**C**) Summary of the time course effects of intraperitoneal injections of saline, 1.0, and 2.0 g/kg EtOH on quinpirole activation of VTA GABA neuron firing rate. Compared to saline injection, EtOH (2 g/kg, IP) progressively and significantly enhanced quinpirole activation of VTA GABA neurons. Asterisks ** represent significance level *p* < 0.01.

Our overall hypothesis is that EtOH interacts with peripheral immune mediators, including MDMs to modulate transmission in the mesolimbic DA pathway. We have recently demonstrated that peripheral D2Rs mediate some effects of EtOH on VTA neuronal responses and DA transmission [21]. Thus, we evaluated the role of peripheral immune substrates, with particular attention to the involvement of peripheral and central D2Rs and MDMs, on dose- and time-dependent effects of EtOH on VTA GABA neurons, DA release, and behavior. We have previously demonstrated that the in situ microelectrophoretic application of DA activates VTA GABA neurons [13,67] via D2Rs and that EtOH modulates the DA activation of VTA GABA neurons [14]. Ethanol had biphasic effects on DA activation, with initial inhibition followed by a trend towards enhancement of activation. Thus, the purpose of this experiment was to determine the impact of acute EtOH on the activation of VTA GABA neurons by the D2R agonist quinpirole over a more prolonged time course. VTA GABA neurons were tested in vivo for sensitivity to quinpirole via in situ microelectrophoresis. Representative quinpirole-activated VTA GABA neurons are shown for both 1.0 and 2.0 g/kg EtOH in Figure 7A,B. As reported previously [13,67], microelectrophoretic application of quinpirole (+30 nA) significantly enhanced the firing rate of VTA GABA neurons (193.3 ± 7.8%; *t*_(45)_ = −1300, *p* < 0.0001, n = 46; mean baseline firing rate = 27.3 ± 2.8 Hz). The effects of acute intraperitoneal EtOH (1.0 and 2.0 g/kg) on the quinpirole activation of VTA GABA neurons were evaluated. Analysis of the baseline (pre-injection) period using a multilevel model revealed no baseline differences in GABA neuron activation between treatment groups (EtOH 1 g/kg: *t* = 0.02, *p* = 0.980; EtOH 2 g/kg: *t* = 0.95, *p* = 0.346). Further, the magnitude of GABA neuron activation was found to be stable throughout the baseline period (time: *t* = 0.27, *p* = 0.786; EtOH 1 g/kg × time: *t* = −0.02, *p* = 0.987; EtOH 2 g/kg × time: *t* = −0.87, *p* = 0.391). Analysis of the treatment (post-injection) period revealed no significant main effects of treatment (EtOH 1 g/kg: *t* = 0.05, *p* = 0.957; EtOH 2 g/kg: *t* = 0.81, *p* = 0.423). Further, for both saline- and EtOH (1 g/kg)-treated rats, the magnitude of D2R-mediated GABA neuron activation did not change with time (time: *t* = −0.92, *p* = 0.363; time × EtOH 1 g/kg: *t* = 0.91, *p* = 0.365). However, for rats in the EtOH (2 g/kg) treatment group, the D2R-mediated activation of GABA neurons in the VTA increased significantly over time (time × EtOH 2 g/kg: *t* = 2.60, *p* = 0.013, *b* = 0.3459 [0.0770, 0.6148], Figure 7C). Thus, these findings suggest that EtOH is producing short-term upregulation in the D2R-mediated activation of VTA GABA neurons. However, this experiment did not resolve if the effect was mediated by peripheral or central actions of EtOH.

### 3.7. Clodronate Reduces MDMs and Blocks EtOH Effects on VTA GABA Neurons and Behavior

**Figure 8:**
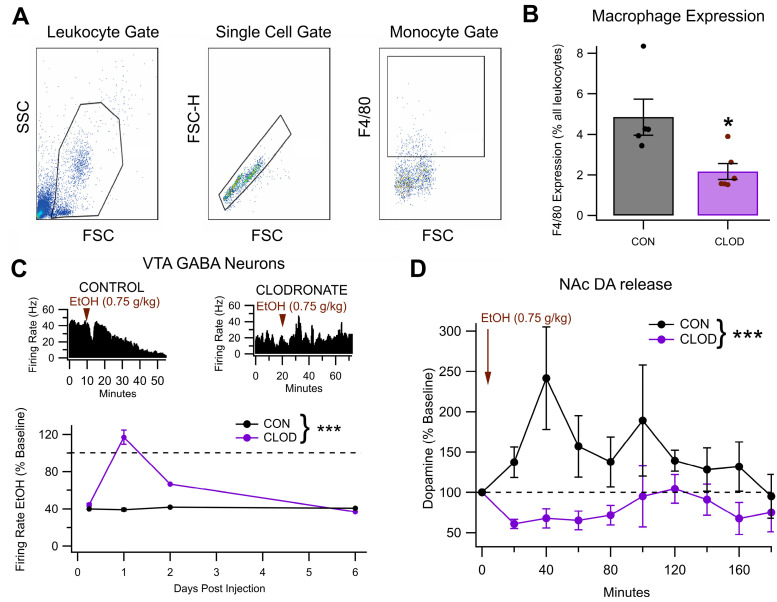
Clodronate Reduces MDMs and Blocks EtOH Effects on VTA GABA Neurons and Dopamine Release in the Nucleus Accumbens: (**A**) The gating scheme for finding D2R expression in monocyte-derived macrophages (MDMs). (**B**) MDMs are reduced in the blood by treatment with liposomal clodronate (CLOD; IV 10 mL/gm) vs. control liposomes (CON) at 24 h after injection as measured by flow cytometry and fluorescent probes for F4/80+ macrophage markers (*n* = 6 and 5). (**C**) Insets show ratemeter record from two representative VTA GABA neurons demonstrating the effects of EtOH at 0.75 g/kg in separate mice injected with CLOD or CON 24 h prior. The graph below shows the time course of CLOD effects on its ability to inhibit the firing rate of VTA GABA neurons produced by EtOH at this dose level. Note that EtOH inhibition of firing rate was approximately 60%, as reported in multiple publications, and the inhibition was sustained in CON-treated mice, but not in CLOD-treated mice. The effect was most dramatic at 24 h after CLOD injection. CLOD significantly reduced the EtOH inhibition of the VTA GABA neuron firing rate (*n* = 5 and 4). (**D**) Dopamine release in the NAc, as measured by microdialysis and HPLC electrochemical detection, was inhibited approximately 40% by EtOH at 0.75 g/kg in CON-treated mice, which was blocked by CLOD. Asterisks *, *** represents significance levels *p* < 0.05 and *p* < 0.001, respectively.

Clodronate liposomes (CLOD) were used to reduce macrophages to measure EtOH effects on VTA GABA neurons, DA release, and behavior. Twenty-four hours post-CLOD injection, the mice showed a significant 55.3% decrease in macrophage expression, as measured by the proportion of F4/80+ cells among all measured leukocytes (Figure 8A,B; *F*_(1, 9)_ = 8.72, *p* = 0.016, n = 6 per group). There was a significant difference in the effects of EtOH on the firing rate of VTA GABA neurons within the first 60 min after a 0.75 g/kg injection of EtOH between the mice that received a CLOD injection 24 h prior and the mice that only received a saline injection 24 h prior (Figure 8B; *F*_(1, 5)_ = 55.38, *p* < 0.001, n = 6 [CLOD], n = 5 [control]). In control mice, EtOH reduced firing to 39.15 ± 1.61% of baseline, while in CLOD mice, EtOH enhanced firing to 116.9 ± 8.76% of baseline. This protocol was repeated at 6 h, 24 h, 48 h, and 6 days post-CLOD injection, revealing that the maximal effect of CLOD on the EtOH-induced suppression of VTA GABA neuron activity occurred at 24 h post-injection (Figure 8C). As mesolimbic DA release is a strong indicator of VTA GABA neuron firing, the effect of CLOD reduction on EtOH-induced DA release in the NAc was tested via microdialysis. Analysis of these data revealed that treatment with CLOD altered the effect of EtOH (0.75 g/kg, ip) on extracellular DA in the NAc (Figure 8D; *F*_(1, 76)_ = 23.26, *p* < 0.001, n = 7 [CLOD], n = 6 [control]) at 24 h post-CLOD injection. Specifically, while in control mice, EtOH enhanced NAc DA levels (*t*_(23)_ = −3.22, *p* = 0.00187), in CLOD-treated mice, EtOH NAc DA levels were reduced from baseline (*t*_(27)_ = 6.600, *p* < 0.0001).

**Figure 9:**
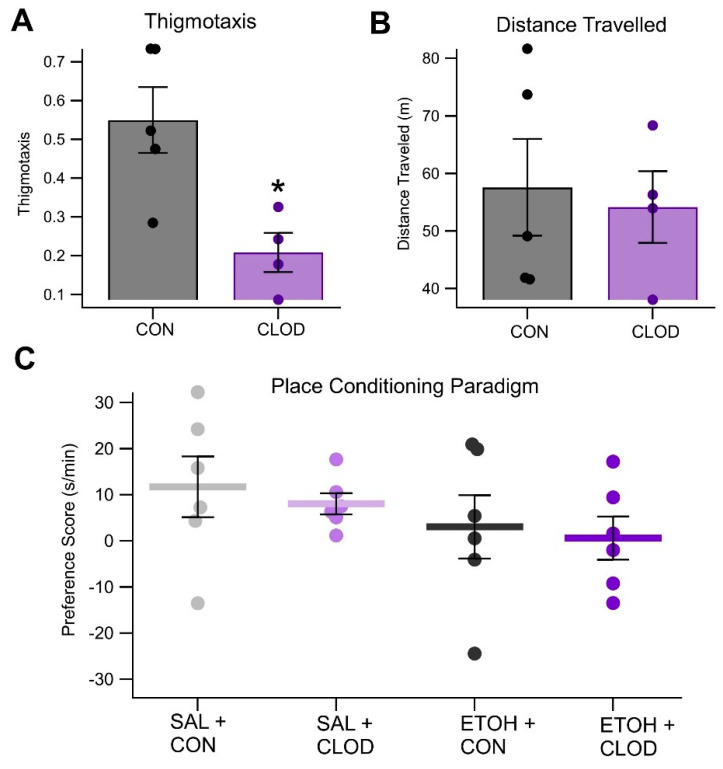
MDM Reduction Modifies EtOH Behaviors: Mice were treated with CLOD or control liposomes (CON) 24 h prior to experiment (IV; 10 uL/gm). Mice were injected with 2 g/kg EtOH and then placed in a large, open cage for 30 min while their activity was recorded with a camera. Video analysis with MATLAB indicated distance traveled and thigmotaxis. (**A**) CLOD-treated mice showed lower thigmotaxis than CON-treated mice after ETOH (*n* = 5 and 4). (**B**) Both groups of mice traveled the same distance after EtOH (*n* = 5 and 4). (**C**) Place conditioning showed no significant effects of EtOH or CLOD. Asterisk * represents significance level *p* < 0.05.

In an open-field paradigm testing behavior post-EtOH injection, the mice that had reduced macrophages (CLOD) showed a significant decrease in thigmotaxis, an indicator of anxiety (Figure 9A; *F*_(1, 7)_ = 10.38, *p* = 0.014, n = 4 [CLOD], *n* = 5 [control]). There were no significant differences in distance traveled between groups (Figure 9B; *F*_(1, 7)_ = 0.067, *p* = 0.80, *n* = 4 [CLOD], n = 5 [control]). In a place conditioning paradigm of EtOH preference, the preference scores revealed no significant effects of either EtOH or CLOD (main effect of ethanol: *F*_(1, 20)_ = 2.21, *p* = 0.1529; main effect of CLOD: *F*_(1, 20)_ = 0.32, *p* = 0.5791; EtOH × CLOD interaction: *F*_(1, 20)_ = 0.01, *p* = 0.9125, Figure 9C). However, analysis of time spent in the treatment context during pre-test and post-test sessions revealed a significant increase in time spent in the treatment context after conditioning (main effect of session: *F*_(1, 20)_ = 4.66, *p* = 0.0432, partial *η*^2^ = 0.1890 [0.0000, 0.4455]). In addition, mice in the EtOH treatment groups spent less time in the treatment-paired context overall, during both pre- and post-test sessions (main effect of ethanol: *F*_(1, 20)_ = 5.16, *p* = 0.0343, partial *η*^2^ = 0.2052 [0.0000, 0.4599]). No other effects were significant (main effect of clodronate: *F*_(1, 20)_ = 0.05, *p* = 0.8319; ethanol × clodronate interaction: *F*_(1, 20)_ = 1.73, *p* = 0.2027; ethanol × session interaction: *F*_(1, 20)_ = 2.21, *p* = 0.1529; clodronate × session interaction: *F*_(1, 20)_ = 0.32, *p* = 0.5791; ethanol × clodronate × session interaction: *F*_(1, 20)_ = 0.01, *p* = 0.9125).

## 4. Discussion

Markers of D2R expression are not only detected in the brain but are also expressed in peripheral tissues, including blood, where DA appears to play a pivotal role in mediating communication between the nervous and immune systems [68], and in particular via D2Rs on lymphocytes [69]. Of particular relevance, D2/D3 receptor expression is known to be altered in a variety of neuropsychological pathologies [22,70,71,72,73,74,75]. It has been suggested that DA receptor mRNA expression in circulating blood might reflect the DA receptor level in the brain [70], serving as a useful surrogate marker for more direct measurements of central receptor status, and by extrapolation, of DA levels in the brain [76]. Thus, this study aimed to evaluate the sensitivity of leukocytic D2R expression, in particular MDMs, and microglia by EtOH and its relevance to VTA neuronal responses, DA release, and behavior.

We first evaluated the effects of EtOH on peripheral leukocyte D2R expression in vivo. We did not know what to expect a priori, as there is little or no literature on this other than our previous study evaluating the effects of DA on D2R expression in lymphocytes and monocytes in RLS [22]. Regardless, these former studies with DA were performed only on lymphocytes vs. monocytes and not on specific populations of lymphocytes and only in vitro, rather than in vivo and in a medicated disease population. Most importantly, we were motivated by our more recent in vivo study demonstrating that peripheral D2Rs were responsible for some of the acute effects of EtOH on midbrain neurons, DA expression, and EtOH behaviors [21]. Thus, we hypothesized that D2R expression in at least some leukocytes would be affected by EtOH. We assessed the effects of three IP doses of EtOH (1, 2, and 4 g/kg) at three time points (0.5, 1, and 2 h) on D2R expression in blood leukocytes. We found that acute EtOH modulates the expression of D2Rs in both lymphocytes and monocytes in a dose-, time-, and type-dependent manner. Acute EtOH significantly enhanced lymphocyte and monocyte D2R expression at 1.0 g/kg by 2 h after injection. It was unclear why EtOH did not have a monotonic dose-dependent effect on leukocyte D2R expression. However, we have reported previously that EtOH enhances DA transmission at 1 g/kg, but decreases DA release at 4 g/kg [21], suggesting that there is something unique about the dose. This is also supported by the lack of consistency in EtOH effects on DA release across dose levels reported by us and others, as mentioned above. Both lymphocytes and monocytes exhibited increased expression of D2Rs with acute EtOH in vivo; however, T cell lymphocytes were not significantly affected, suggesting some specific effects by lymphocyte type. However, while lymphocytes were unaffected by EtOH/DA/EtOH+DA in vitro, EtOH decreased monocyte D2 MFI in the presence of DA, while increasing the percentage of cells expressing D2Rs in vitro, suggesting involvement of an additional signaling factor or possibly an EtOH metabolite. These data demonstrate that acute EtOH not only alters peripheral immune cell signaling but may also initiate downstream effects on the CNS through D2R-expressing monocytes and lymphocytes. Given previous evidence that peripheral D2Rs influence central DA signaling [21], we next examined how this peripheral immune activation may contribute to central neuroimmune responses and neuronal function.

It has been found that microglial activation and subsequent neuroinflammation might be involved in the neurodegeneration and cognitive dysfunction produced by chronic EtOH use [77,78]. To test whether this peripheral immune activation is linked to changes within the CNS, we evaluated microglial responses—specifically D2R expression and inflammatory polarization—in the mesolimbic system. It has been well established that microglia express DA receptors, including D2Rs [79]. These D2Rs have a modulatory role in neuroinflammation [80]. To determine if acute EtOH induced changes in microglia morphology, we imaged microglia in the NAc and VTA of MaFIA mice injected with EtOH (1, 2, or 4 g/kg, ip) and euthanized at 0.25, 0.5, 1, or 2 h post-injection. No significant changes to morphology due to EtOH reactivity were detected. However, it was found that there were regional differences in morphology between the NAc and VTA. It is important to note that in the study of morphology some time/dose combinations had ≤3 animals, so it is possible that the activation trend could continue if we had more power in that group. As an additional refinement on this method, we decided to examine the polarization state of the microglia. The microglial activation state has a well-established influence on cell function and metabolism. Classically, M1-like activation is associated with pathogen and damaged tissue clearance, while M2 polarization is associated with tissue repair and immune regulation [33]. Although both states can involve similar morphological changes, function polarization provides additional insight into the role of microglia in neuroimmune responses. Consistent with the hypothesis of LPS-induced activation, we observed a shift toward a proinflammatory (M1-like) activation in mesolimbic microglia following acute EtOH. M1-type microglia release inflammatory cytokines such as TNFα, MCP-1, IL-1β, and IL-6 in response to LPS and other noxious stimuli, and these cytokines may be responsible for acute or long-term effects of EtOH exposure. Therefore, these cytokines may be useful targets for future treatments. It is important to note that while M1 vs. M2 are no longer considered sufficient to define microglial phenotypes, their use was consistent with accepted practices at the time of data collection. Despite this limitation, the observed shifts in CD40 expression provide meaningful insight. Although the effects on striatal microglia polarization and D2R expression in the NAc are robust, it is interesting that no significant changes to morphology were observed. We show changes to microglial D2R expression and polarization in response to acute EtOH that provide evidence for the importance of these striatal microglia in the neuroimmune response to EtOH. Here, we show that acute EtOH resulted in increased D2R expression on microglia in the NAc. This same result was not found in the VTA, indicating regional differences in microglial EtOH response. We recognize that the dose level of EtOH used to evaluate microglial morphology and D2R expression was at a higher dose of EtOH (2.0 g/kg) than that which enhanced monocyte D2R expression (1.0 g/kg). Unfortunately, the IHC experiments at 1.0 g/kg were not usable because of antibody issues. We could not justify another cohort of animals at this dose. But, we feel that 2.0 g/kg is still in the justifiable range.

During withdrawal from chronic EtOH, critical DA-related gene products, such as tyrosine hydroxylase (TH) are upregulated [81], while D2Rs are downregulated [82]. This decrease in D2R expression, however, is accompanied by increased D2R function. In the present study, we found that acute EtOH enhanced the D2R-mediated activation of VTA GABA neuron firing. Preliminary evidence for this was described in our previous study [14], but the enhancement was only followed for less than 1 h, and there was only a trend in enhancement at 1 h post-injection. We replicate the phenomenon here, but with the agonist quinpirole for D2R selectivity and with a time course up to 4 h. The progressive enhancement of D2R activation by acute EtOH at 2 g/kg could indicate adaptations in D2R sensitivity due to local circuit effects or the result of neuroimmune interactions. Dopamine and D2R agonists typically activate GIRK-mediated D2R autoreceptors on DA neurons. However, we have shown previously that D2R transcripts are also expressed in VTA GABA neurons, but at a much lower level of expression as DA neurons [14]. The in situ microelectrophoretic application of DA enhances the VTA GABA neuron firing rate, but also couples spikes via Cx36 gap junctions (GJs) [67]. Indeed, DA activation during the iontophoretic ejection current slows because of this coupling, which is blocked by EtOH [13]. Thus, we decided not to pursue more mechanistic studies to resolve this here, as the mechanism for the DA or D2R agonist activation of VTA GABA neurons is still not resolved. Future studies are necessary to unravel the role of GJs in DA activation, and/or the role of D2R desensitization at the central and peripheral levels. However, the time course of the enhanced D2R-mediated activation of VTA GABA neurons following EtOH administration closely parallels the peripheral upregulation of D2Rs in monocytes and microglia polarization, suggesting a potential mechanistic link between peripheral immune activation and central DA circuit modulation.

Though the primary immune cells of the CNS are microglia, other myeloid cells such as MDMs often accompany inflammation [83]. CNS macrophages exhibit many different functions within the CNS, including the clearing of debris and regulating neuronal firing. Often, these macrophages express chemokine receptors and integrins that influence the positioning of microglia throughout the CNS [84]. Additionally, chronic EtOH is linked to the activation of tissue macrophages [85]. These peripheral macrophages have been shown to have increased secretion of proinflammatory cytokines in active EtOH consumers [86,87]. Therefore, a relationship between EtOH and MDM could be suggestive of a neuro-immunomodulatory effect. To directly assess whether peripheral immune cells mediate the central and behavioral effects of EtOH, we used liposomal clodronate to selectively reduce circulating MDMs. If MDMs contribute to CNS modulation, their reduction should attenuate EtOH’s central effects—which is precisely what we observed. In our experiments in a macrophage-reduced model of acute EtOH exposure (i.e., MaFIA mice), the normal responses to EtOH, including suppression of the VTA GABA neuron firing rate, DA release in the NAc, and open-field indices were greatly reduced. We acknowledge that the dose of EtOH used in DA release experiments (0.75 g/kg) was lower than the dose level used to evaluate D2R expression in leukocytes (1.0 g/kg). This was mostly due to previous experience with EtOH on DA release in mice. We have reported previously that the EC_50_ for the EtOH enhancement of DA release was 0.75 g/kg [21]. Thus, we chose to evaluate the role of MDM depletion at this dose level of EtOH to stay within the dynamic range. This indicates the relevance of these peripheral macrophages and the inflammatory cytokines they produce in acute EtOH response. Others have shown that inflammatory cytokines affect the firing of neurons throughout the brain [88]. Surprisingly, although there was not a significant effect of either EtOH or clodronate on the induction of conditioned place preference, mice spent significantly more time in the treatment context during the post-test session. This may indicate that repeated exposure to the initially unpreferred context reduced its aversiveness.

Finally, monocytes that express DA D2Rs could be activated by circulating DA levels during EtOH administration, which may provide a signal for increased or decreased infiltration of the CNS. Further experimentation is required to examine what is happening during withdrawal to these myeloid cells that are present in the gut and in the blood. While MDM D2R expression was affected by EtOH more than other leukocytes, and MDM depletion abolished the effects of EtOH on peripheral and central EtOH effects on mesolimbic substrates, a more mechanistic experiment needs to be performed to determine more definitively if monocyte D2Rs mediate the effects of EtOH. We considered a D2R knockout model, but there are major vagaries associated with this model, not to mention that developmental adaptations preclude mechanistic determinations. A plausible mechanistic experiment that we have considered would be to cross C57BL/6-Ccr2em1 (icre/ERT2)Peng/J mice with B6.129S4 (FVB)-Drd2tm1.1Mrub/J to induce selective deletion of monocyte D2Rs with tamoxifen. However, this method only just became available. Additionally, going forward it will be interesting to examine how macrophage or microglia affect drinking behaviors and EtOH seeking in mice. An understanding of EtOH’s effects on the innate immune system will help us to better tailor treatments for AUD patients. Microglia and MDM represent a unique and interesting target for potential therapies. Human monoclonal antibodies are already in use to block the effects of other immunological diseases and could potentially be adapted to regulate EtOH-induced peripheral neuroimmune activation without modulating central processes directly. In addition, other immunosuppressant drugs such as the antibiotic minocycline inhibit myeloid function and could potentially be used to block these EtOH effects. An understanding of the brain’s innate immune system could contribute to a better understanding of patients and future treatments for AUD.

## 5. Conclusions

**Figure 10:**
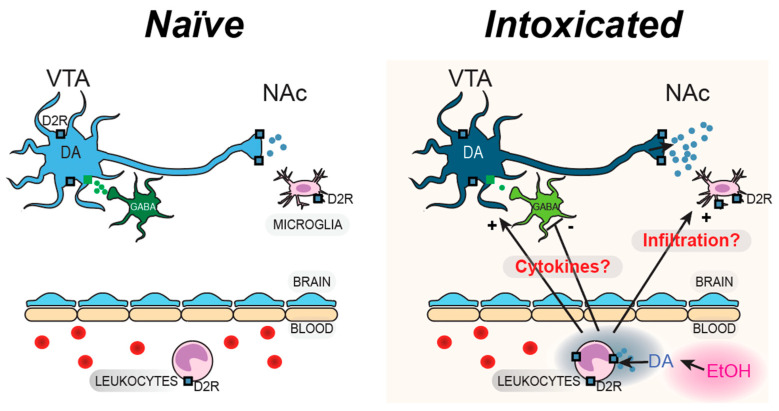
Theoretical Framework for Neuroimmune Effects of Acute EtOH: In the EtOH-Naive condition, DA neurons in the VTA project to the NAc via the medial forebrain bundle. Dopamine neurons are inhibited locally by VTA GABA neurons, as well as indirect feedback circuitry from the NAc and other brain regions. In the EtOH-Intoxicated condition, acute EtOH inhibits VTA GABA neurons [4,13,14], thereby disinhibiting VTA DA neurons and enhancing DA release in the NAc and EtOH reward. However, we propose that this results, in part, from an initial action in the periphery via D2R-expressing leukocytes. The model proposes that activation of peripheral D2R-expressing leukocytes by EtOH or by blood DA levels enhanced by EtOH [21] activate D2R-expressing microglia, either by infiltration or by cytokine release in the periphery [89]. Darker shading of neurons and thickness of axons indicates more excitability.

Taken together, these data suggest that there is a strong peripheral neuroimmune component to the body’s reaction to acute EtOH, even at mildly intoxicating doses. We found that in the periphery, D2R expression is enhanced on lymphocytes and monocytes with acute EtOH and that the reduction of peripheral macrophages inhibited the typical behavioral and electrophysiological effects of EtOH. Centrally, we found upregulation of proinflammatory markers on striatal microglia and an increase in D2R expression on these microglia in the NAc. In the context of the sensitization of D2R receptors to activation by EtOH over time, we see that these peripheral and central neuroimmune factors may mediate their role in the EtOH response through D2R receptor activation and sensitization. The purpose of this project was to investigate the neuroimmune interactions of the innate immune system following an acute injection of EtOH. More specifically, the activation and interaction of peripheral leukocytes like lymphocytes and monocytes and CNS microglia were investigated, with particular attention to peripheral and central D2Rs. We present a theoretical framework model wherein EtOH causes leukocyte infiltration of the BBB and activation of mesolimbic microglia (Figure 10).

The inflammatory response is in part responsible for the changes to GABAergic transmission to DA neurons, DA release, and the rewarding properties of acute EtOH. It is uncertain whether this results from brain infiltration of MDMs or the release of cytokines peripherally or centrally. Thus, release of cytokines may be a more logical interpretation. Indeed, we have recently shown that the anti-inflammatory cytokine IL-10 increases DA neuron activity and release [89]. Together, these findings build a compelling case for a functional pathway where EtOH-induced peripheral immune activation, marked by changes in D2R expression and MDM activity, drives central microglial activation and alters mesolimbic DA circuit function. We acknowledge that these peripheral neuroimmune effects of EtOH do not account for all the effects of EtOH centrally, as there are many receptor systems implicated in EtOH central effects, including the enhancement of GABAergic synaptic transmission that we and many others have reported. However, in our hands, EtOH does not affect the activity of VTA GABA neurons ex vivo, but markedly affects them and their GABAergic adaptation in vivo. Thus, we were influenced by years of frustrating negative studies on VTA GABA neurons ex vivo, and posited that peripheral effects might be involved. This study provides foundational evidence for future studies investigating the role of neuroimmune interactions in acute EtOH response and potential therapies based on this connection.

## Data Availability

The pre-clinical data supporting the findings of this study are available from the corresponding author upon reasonable request.

## Supplementary Materials

The following supporting information can be downloaded at: https://www.mdpi.com/article/10.3390/biomedicines13102327/s1, Video S1: Neun IBA-1 3D projection of Microglia and Neurons in the VTA.

## Abbreviations

The following abbreviations are used in this manuscript:

AUC	Alcohol use disorder
VTA	Ventral tegmental area
DA	Dopamine
NAc	Nucleus accumbens
EtOH	Ethanol
GABA	γ-aminobutyric acid
D2Rs	Dopamine 2 receptors
NK	Natural killer
BBB	Blood–brain barrier
CNS	Central nervous system
TLRs	Toll-like receptors
MDMs	Monocyte-derived macrophages
MaFIA	Macrophage FAS-Induced Apoptosis
IP	Intraperitoneal
PBS	Phosphate-buffered saline
PFA	Paraformaldehyde
BSA	Bovine serum albumin
RLS	Restless legs syndrome
MFI	Mean fluorescent intensity
IHC	Immunohistochemistry
CLOD	Clodronate liposomes
